# Making a livelihood at the fish-landing site: exploring the pursuit of economic independence amongst Ugandan women

**DOI:** 10.1080/17531055.2013.841026

**Published:** 2013-09-26

**Authors:** Georgina Pearson, Caroline Barratt, Janet Seeley, Ali Ssetaala, Georgina Nabbagala, Gershim Asiki

**Affiliations:** ^a^ Department of International Development, London School of Economics and Political Science (LSE), London, UK; ^b^ School of Health and Human Sciences, University of Essex, Wivenhoe Park, Colchester, UK; ^c^ MRC/UVRI Uganda Research Unit on AIDS, Entebbe, Uganda; ^d^ UVRI-IAVI, Entebbe, Uganda

**Keywords:** women, gender relations, fisheries, livelihoods, Uganda, Africa

## Abstract

Qualitative life history data were used to explore the experiences of women who live at five fish-landing sites on Lake Victoria, Uganda. We explored what economic and social opportunities women have in order to try to understand why some women are more vulnerable to violence and other risks than others and why some women are able to create successful enterprises while others struggle to make a living. The ability of women to create a viable livelihood at the landing sites was influenced by a wide variety of factors. Women who had or were able to access capital when they arrived at the landing site to set up their own enterprise had a significant advantage over those who did not, particularly in avoiding establishing sexual relationships in order to get support. Being able to establish their own business enabled women to avoid lower paid and more risky work such as fish processing and selling or working in bars. The development of landing sites and the leisure industry may be having an impact on how women earn money at the landing sites, with the most desirable economic opportunities not necessarily being connected directly to fishing.

## Introduction

Women in fisheries in Africa have received increased attention in recent years, with a complex picture emerging of their valuable contribution at landing sites as, for example, fish processers, fishmongers, restaurant and bar workers, as well as mothers and partners.^1^ However, given the negative portrayal of fish-landing sites in many settings as ‘immoral spaces’ because of a youthful and relatively free population engaged in risk-taking, alcohol use and prostitution,^2^ women at these sites have been seen in the literature as passive victims of this culture engaged in transactional sex.^3^ Tindall and Holvoet^4^ argue that the fisheries sector has been slow to move away from a view of women as processers and men as users and managers of the resources. Dispensing with this simple dichotomy and appreciating the complexity of gender relations within the context of fishing communities in Africa requires an understanding of where women are able to negotiate and create opportunities for themselves. In this paper we use qualitative life-history data to explore the experiences of women who live at five fish-landing sites on Lake Victoria, Uganda. We explore what economic and social opportunities women have in order to try to understand why some women are more vulnerable to violence and other risks than others and why some women are able to create successful enterprises while others struggle to make a living. We start with a brief review of previous work about women at fish-landing sites in Africa and outline the theoretical framework for our analysis.

### The gender division of economic activities in fishing communities

The lack of economic opportunities for women at fish-landing sites in Africa has been described as a key contributing factor in the vulnerability of women in fisheries.^5^ Mojola^6^ neatly summarized this as ‘men fish and women sell fish’. However, the economic vulnerability of women does not only result from the disparate profit generation between these two activities; it also emerges from the ability, or lack thereof, of women at the landing site to negotiate access to the fish to sell. Geheb et al.,^7^ in a study of gender in Lake Victoria fisheries, note that women who were involved with fisheries tended to work on the periphery, as they were largely unable to engage with the capture fishery which men dominated or to compete with factories which were able to offer much higher prices. As a result they were limited to buying and selling fish not required by more powerful industry actors, thus making the activities to which they had access even less profitable. Lack of access to credit also made it very difficult for women with capital to get started in business; whilst lack of access to land on some landing sites meant that women were unable to provide for their subsistence needs without acquiring cash.

Furthermore, increased competition in the fishing industry where there are dwindling fish stocks, or shortages of particular types of fish, means that as more men become fishers, they may find it increasingly hard to find an entry point into the capture fishery and so start to trade or process fish. Medard^8^ notes that women in the *dagaa* (small silver fish; *Rastrineobola argentea*) fishery on Lake Victoria in Tanzania were being pushed out by men seeking to diversify their options as the Nile perch fishery, on which they had been dependent, declined.^9^ Medard describes an additional risk factor for women's engagement in fisheries: many of the women's activities described in her study were considered illegal due to the type of nets that they used, which made them vulnerable to enforcement action.

In some places the capture fishery has been off-limits to women for both economic and social reasons. Madanda^10^ reports that many cultural beliefs and social norms prevent women from engaging in fishing on Lake Victoria; in one study she found that it was widely believed to be bad luck to meet a woman on the way to the lake and that if you have a woman on the boat you will get a low catch. Nevertheless, Madanda^11^ also reports that some women were directly involved in fishing and that some of the stereotypes were being challenged by women.

### Transactional sex in fishing communities

The exchange of sexual services in order to obtain access to fish by female fish processers and mongers, so-called ‘fish-for-sex’, has been documented in some sites in Africa. Merten and Haller,^12^ for example, maintain that fish-for-sex exchanges in the Zambian Kafue Flats are a result of the lack of income-generating alternatives for women. However, rather than being a simple manifestation of the poverty and vulnerability of female fish traders, Béné and Merten^13^ suggest that this practice is carried out by social actors who have at least some power to negotiate. However, the picture is mixed, because Kwena et al.,^14^ in their research in Kenya, found that female traders did not have the bargaining power to refuse such exchanges or to negotiate for safer sex practices.

Although fish-for-sex has received attention in the literature, other forms of transactional sex do exist at landing sites and in many other settings. This form of exchange is defined by Mojola^15^ as:

non-marital, non-commercial sexual relationships where money and gifts are exchanged, but in which issues of love and trust are sometimes also considered at stake. The predominantly one-way transfer of money and gifts – from men to women – reflects the fact that in most settings, men have greater access to money and resources due to the gendered structure of local economies.

Pickering et al.^16^ found that women in a fishing community on Lake Victoria reported nearly always receiving gifts or money in return for each sexual contact. Regular partners tended to be less likely to give money than casual contacts, but many gave in-kind support. Only 35% of contact with regular partners resulted in remuneration in cash compared with 77% of contacts with casual partners. Only 1% of casual and 5% of regular contacts did not result in the woman being given cash or gifts.

Research that does not focus solely on fishing communities indicates that transactional sex is a widespread practice and that women can get a sense of power from exploiting their sexuality,^17^ a narrative that has been missing in some of the literature on sex-for-fish.

### Women's economic independence

Obbo argued in 1976 that ‘Although an asymmetric public power relationship generally exists between the sexes in Africa, women are, and have always been, improving their position individually within their respective, societal frameworks’.^18^ However, the economic independence of women may be viewed with suspicion because of the perceived neglect of family duties and the threat of their economic freedom to gender norms in patriarchal societies, such as the Baganda, the dominant tribal group in central Uganda. Davis^19^ writing about women in Kampala (the capital city of Uganda), for example, argues that economically independent women are portrayed as not having time to fulfil family roles such as looking after children and family; while Godfrey,^20^ also writing about women in Uganda, observed that women's increased involvement in monetary activities was not without ambiguity: empowerment being seen as transgressing or challenging social norms.

Economic independence amongst women has also been associated with prostitution. Davis^21^ described the stigma that may be associated with economically independent women, arguing that the liminality of ‘town women’ in Kampala, who were often equated as being ‘prostitutes’, was in part due to this economic independence they had achieved. Similarly, Kuhanen^22^ noted that ‘prostitution’ was a label used for single women (whether divorced, widowed, abandoned and those who chose not to have a husband) who were economically independent.

Whilst the informal economy has opened up livelihood opportunities for some women at fish-landing sites, this is not always the case. By exploring the economic and social aspects of women's experiences at the landing sites, this paper illustrates how opportunities are not easily reached by all, are often shifting and come with not just socio-economic risks, but also risks to safety and to health.

We now describe the case study communities where the research was carried out and the methods used to collect and analyse the data.

## Methods

Life-history data were collected by the Uganda Virus Research Institute (UVRI) as part of a prospective cohort study from 2008 to 2010 looking at risk factors for HIV infection in five fishing villages and landing sites in Masaka, Mukono and Wakiso districts on the shores of Lake Victoria.^23^ For this qualitative component of that study, we purposively selected participants to reflect different age groups, occupations and sexes from a sample of volunteers who were screened out of the main epidemiological study where an HIV-negative cohort of 1000 people was enrolled. We recruited participants from a group of volunteers who were ineligible for the HIV-negative cohort because of their HIV-positive status at the time of enrolment, as well as from volunteers who enrolled but later sero-converted during follow-up and voluntarily disclosed their status and indicated their willingness to participate in other studies.^24^

The epidemiological study and qualitative sub-study received ethical approval from the Science and Ethics Committee of the UVRI and overall approval from the Uganda National Council for Science and Technology. All participants gave written, informed consent.

Seventy-eight women and men agreed to take part in two or three life-history interviews about their childhood, family life and sexual relationships. In addition, 40 people from different interest groups at the sites (bar owners, boat owners, local officials, for example) were interviewed as key informants. Experienced local fieldworkers conducted the interviews over several weeks and then compiled each interview with an individual into a single life-history narrative. Fieldworkers and participants were matched by gender, except in one case where one older woman interviewed several male participants; this was because of this fieldworker's exceptional interview skills and good rapport with the community which made men more willing to talk to her than to a man of their own age. Interviews were conducted in a private location. Tape recorders were not used because participants were concerned about confidentiality and privacy. Interviews were conducted in Luganda by native speakers and written up in English by the interviewer. Luganda was retained in the transcript for particular phrases and comments mentioned by the interviewee. Translations of idioms and proverbs (commonly used in Luganda) were discussed among team members to agree on the translation. All identifying information, including names, was removed from the interview transcripts and an identification number used instead.

Two team members, Ugandan and British, analysed the data by first reading all the interview transcripts and selecting themes to use for coding, which were discussed and agreed among the research team. All the data were then coded manually. During the analysis the clarification of meanings within the narratives was discussed among the team members. The data were subsequently analysed in three main stages. Firstly, a thematic analysis of the life histories and key informant interviews was undertaken with open coding to detect emerging themes. The main themes related to the informant's background, lifestyle and livelihoods, and perceptions about HIV/AIDS, then after discussion on the emerging findings further analysis focused on the women at the landing sites.

The second part of the analysis therefore involved further exploration of the 30 female life histories and 19 female key informant interviews. Using an inductive approach^25^ emerging themes and patterns were identified and explored and case studies purposively identified to illustrate patterns within these themes. The patterns clustered around broader themes, with the main ones explored in this analysis being: background of the women at the landing sites; factors relating to their decisions to move to the landing sites; and lifestyle and livelihoods at the landing sites, especially the risks and opportunities faced relating to economic activities and sexual relationships.

The focus of this paper is on women's experiences at the landing sites with the majority of the analysis based on female life histories and key informant interviews. However, the male life histories and key informant interviews were also drawn upon for the context and to build a broader understanding of experiences at the landing site.

## Findings

### The setting

Many of the fish-landing sites on Lake Victoria have grown up over the last 30 years in the wake of a boom in Nile perch fisheries, which reached a peak in the 1990s, attracting many young people to the lakeshore in search of a cash income from fish or fish trading and associated activities.^26^ While the boom has now passed many young people still come to the fish-landing sites on the lake shore and on the islands in Lake Victoria. All the landing sites are ethnically mixed as people come from all over Uganda and some from neighbouring countries in the hope of making a living and, often, an independent lifestyle away from family ties.^27^

In all five landing sites in our study many different activities were carried out. Men were involved in fishing, loading and off-loading boats, fishmongering and repairing nets, whilst women were involved in fish processing, fishmongering, market vending, and restaurant and bar work. Whilst both men and women owned bars, they tended to be run by women. The landing sites studied varied in terms of their amenities with areas set aside for fish processing, markets and small restaurants. Some had lodges and petrol stations, and were served by local minibus taxis. As some landing sites had developed and become places of more permanent residence, three had primary schools for those children growing up there. The fish were sold locally, or put in ice and packed for transport to fish factories for export.

Some of the men and women moved between the different landing sites and islands on Lake Victoria with up to 47% of men and 25% of women reporting being away from home for at least two days in the previous month.^28^ This was mainly driven by the fish catch which is seasonal, leading to fishermen moving between sites, sometimes being away for three to six months at a time. Longer-term changes in the scale of fishing activities have also had an impact – in fact one landing site is now predominantly based around timber work rather than fishing because of declines in fish catches, which has affected the livelihood options of women as well as men.

There were a large number of bars at the landing sites and within each landing site leisure was seen as revolving around alcohol and sex. As one key informant said:

People outside the fishing community regard us the fishing community as badly behaved. We are regarded as people that having misbehaved elsewhere came to the fishing community as the last resort, running away from legal acts against us. However, it is true that the fishing community is regarded as behaving recklessly; more especially their sexual behaviour is really bad, people here are careless with their sexual life; indeed the promiscuity here is so high. The kind of life style here is bar life mostly for men while female new comers start their life with bar work, bistro work and sex worker. Men are fond of alcohol and sex all the time. We are not respected at all!  (56-year-old female, local official)

### Choosing ‘fishing’ as a living

Many of the women at the landing sites had not been born there. They, like many of the men who go to the sites for work, had left their home villages and family and set up life at landing sites where they may, at least initially, have had relatively little support. The women who made the move to a landing site came from diverse backgrounds, but there were common themes that emerged through their life histories.

The majority had not completed secondary or primary school. For some this was because of a lack of funds, whilst others became pregnant and had to drop out of school. A minority left school due to other external factors such as civil conflict. At the time of moving to the landing site many had children to support from previous or current relationships. Information from two of the female respondent's about their lives prior to moving to the landing site illustrates some aspects of this.

#### Twenty-two-year-old female; sun-drying fish

The respondent's parents died when she was two years old and she was brought up by her maternal grandmother. She stopped education in primary seven (the final year of primary school) due to a lack of funds. Following this she stayed with a cousin who subsequently advised her to get married which the respondent agreed to: ‘there is nothing to do since there is nowhere to get the school fees to continue my studies’. The husband paid brideprice to the cousin of 5 kg of salt, 5 kg of sugar and 12 sachets of *mukwano* (a local brand) tea leaves. The respondent became pregnant twice, but neither child survived infancy. With her inability to give birth to a healthy child, her in-laws described her as a ‘bad omen’ and so she separated from her husband, returning to her family. Subsequently, her aunt's daughter told her to come and stay with her at one of the landing sites where she owned a bar.

#### Thirty-five-year-old female; sun-drying silver fish

The respondent's father was polygamous and her mother was his last wife. In the final year of primary school she became pregnant by another pupil, but the father of the child left for another school where he took another wife. The respondent's child was looked after by the father, but when she heard that the child was being mistreated by the stepmother, the respondent took her child and went back to look after her own sick mother. It was here that a village friend, concerned that she was not working, suggested she got work at a landing site.

Six women reported that they followed their husband or partner who had moved to the landing site for work. This was sometimes a joint decision, at times there were few alternatives for the woman if she was going to sustain an economically supportive relationship. For other women, like the two in the descriptions above, it was not a male partner but female friend or family member who encouraged them to move to the landing site. As in the first case, a female friend or relative who owned a bar or eating place at a landing site would ask them to come specifically to work for them, often providing accommodation. Only four of the 30 women moved on their own, not directly influenced by another person, although two of these women had previously had contact with people at the landing site.

Migration has long been a livelihood strategy when deepening rural poverty, alongside increased freedom of female movement, has led women to seek opportunities in the developing urban centres. Obbo noted that in rural–urban migration, women often draw on ‘networks of friends, relatives and acquaintances’.^29^

However, the move to the landing site was not always smooth. Some women moved quickly to get away from problems, such a marital breakdown or conflict with their family, or went to the landing site because circumstances meant they needed to earn a living and there were no options where they were staying. For example, a 29-year-old female reported that she initially moved to the Lake Victoria area when her husband was stationed at the local barracks. When their marriage broke down she moved to a nearby landing site where she had friends and was able to earn a living doing household work such as cleaning, ironing and fetching water. Through this and keeping pigs she had managed to send her children to school.

A common theme that runs through the women's narratives was that prior to moving their relationships with family, husbands, partners, in-laws or co-wives were problematic. A number described ‘mistreatment’; a few described more overt violence. The following synopses illustrate these aspects.

A 37-year-old woman who sundries fish said she was one of 11 siblings. She stopped formal education at primary three (the third year of primary school) due to a lack of funds, and worked in her mother's garden. She married a soldier, who paid brideprice, and to whom she was married for 12 years and had four children. The husband married a second wife and they lived in the same compound. The respondent reported that the co-wife burnt one of her babies out of jealousy over the husband. She went to the police but no further action was taken. The baby later died. She asked her husband for a separate house; however, he refused and following this they separated. She then met her second husband who she started working with. They were not legally married. They had two children together. During her second pregnancy this husband left for the landing site and later asked her to join him there.

Another respondent, a 47-year-old woman, suffered violence from her father. Her parents, small-scale farmers, had 25 children, of which 23 died. The respondent left education at primary two due to lack of funds. She described her father as a ‘drunkard’ who used to beat her mother. Her parents divorced and her father remarried and had two further children, whilst her mother, who later died, moved to a landing site and worked in a restaurant. The woman reported that her stepmother would accuse her of ‘falling in love with men’ and so her father beat her for her behaviour. She therefore left to stay with her sister, who on noticing her talking with men decided the respondent should be married. When their father came, he called the police to stop any marriage, but the prospective husband had the brideprice ready to pay so the marriage planned by her sister went ahead. They had 14 children together, four of whom died. They were married for 25 years, and during this time the husband married a second wife. He had wanted female children and had only had male children with the respondent at the time (it is not clear if the woman also wanted more children). When the second wife died he returned to the respondent. However, when the husband became sick she suspected he was infected with HIV as his second wife had already died; she therefore separated from him and left for her father's place. It was from there that her sister took her to one of the landing sites so she could earn money.

These examples highlight that whilst the inequalities faced by women at the landing sites are great, as illustrated in other studies,^30^ the women in this study had faced significant inequalities in their lives before moving to the fishing site. Many had limited formal education and were driven by financial hardship and adversity. While the landing site looks like a place of limited opportunities for women, for many women other places have little to offer, especially for those without supportive partners or parents.

In Obbo's case study of single, unskilled female migrants in Kampala, she describes how one woman gradually accumulated capital and built up businesses, moving stage by stage from the village, to a rural centre, to a township and then on to Kampala, where she established a profitable business. In this way, some women were able to be successfully economically independent – success they attributed to ‘toughness and luck’. There are interesting parallels and contrasts with this earlier work on rural–urban migration and the material presented here. In the life histories of women at the landing sites, for many the landing site was the first place they had come to after leaving their natal or marital home and they then stayed on for some years trying to make money; others had tried a few avenues before moving to the landing sites. Whilst some women managed to create relatively successful economic opportunities at the landing sites, others saw it as a temporary stage, hoping to move on or back to their family yet became caught in a struggle to accumulate enough capital to do so.

The women came to the landing site seeking employment and money in order to support themselves and their families, as well as, in some cases, for their own independence. Men often started with activities such as loading and off-loading fish and other cargo from boats, while many women began life at the landing sites in either bar or restaurant work or in some sort of fish-processing job. Most jobs at the landing sites for women involve daily cash incomes, which were unpredictable because of the vagaries of the fish catch. Access to immediate cash has been associated with alcohol use and commercial sexual activity at fishing sites and in other settings.^31^ The next section shows how access to cash for women may have an ambiguous effect on women's bargaining power.

### Fishing-related activities

One avenue for women to make an income is in fish processing. Many women may start by buying one tin (about the size of a bucket) of small silver fish which they sun-dry and with the profit they make from selling them they buy further stock. In this way they may slowly build up their business. Sometimes this activity has involved their male partner who is a fisherman and supplied them with the fish to dry and sell on. It can be hard work and produces little profit at the end of each day, so while some women were able to build up their business, others struggled. For example, a 19-year-old woman had worked in a bar and processing fish, but because of low fish stocks was not able to get work at the time of the interview. She had had sexual partners who offered support at times, including her current partner who was a fisherman. However, support from partners was not reliable because their own work was unreliable and their income inconsistent.

#### Retail, tailoring and restaurants

Other women set up relatively successful businesses such as tailoring, restaurants or market vending. A 31-year-old woman used her previous training in tailoring to set up her own successful business at the landing site. She left education in the second year of secondary school when she became pregnant. The father of the child agreed to care for the child and pay for her to return to education once the child was born. She was therefore able to attend a tailoring course and was given capital by the father of her child to start up her business at a local market, expanding to five small stalls of second-hand clothes. The child was looked after by both her mother and the father's mother during this time. Unfortunately, all her stock was stolen and she had to start the business again. At this point the father of their child stopped providing support for her, although she still had the house they had shared. She therefore rented out that house and, on a suggestion from a friend, moved to a landing site. There she had set up a tailoring business. Initially a female friend paid for her rent, but she saved money to buy her own room.

This woman had been able to recover from shocks as they occurred because of the support she had received as well as her training. Some women did not have this background and used other strategies to start a business. Some farmed, growing food for their own consumption, and used the excess produce either to sell at market or to prepare for selling in a restaurant. This is illustrated in the following account.

A 36-year-old woman moved to the landing site with her husband who would bring back fish and vegetables for her to sell. Later she started to buy her own fish to sell on. After her husband died, she had another partner who she joined in farming, but the relationship did not last. Following their separation, she continued farming and started a cooked food business with the excess produce.

#### Bar work

Bars were places that fishermen went to for entertainment and to spend their money having been on the lake overnight. Female bar attendants were usually between 14 and 20 years old. Bar work, like most activities at the landing sites, is dependent on the fish catch; when the fishermen have no catch to sell they cannot spend their money in bars and restaurants.

A 27-year-old woman initially worked in her uncle's bar when she came to the landing site. Following a number of pregnancies and periods of illness she returned to bar work, until another family member gave her a sum of money that she used to buy a plot of land and construct a house. She started to run a bar from her house; however, with declining custom she moved into market vending.

Many women engaged in bar work complained about the scarcity of customers either because fishermen moved to other fishing sites or because of competition between the increasing numbers of bars at the landing sites. Other concerns included: low wages which meant that they needed to supplement their income; customers fighting or quarrelling or not settling their payments; and ‘disturbance’ from male customers wanting sexual relationships with them. Key informants, both male and female, spoke of these challenges for women too. They perceived that under the influence of alcohol, poorer choices were made by women about sexual partners; there were quarrels over and between partners; and there was risk of violence between men and women, including rape.

Many women had partners who were customers at the bar or restaurant where they worked. The majority of these customers were fishermen, but some were fish truck drivers and younger males doing manual jobs at the landing sites.

When you are working in a bar; it takes no minute to get a sexual partner once you wish for it. … Fishermen are the majority of the customers that come along for booze in bars at the landing sites; and of course they are the likely sexual partners that we get.  (27-year-old female)

Bar attendants were not necessarily paid, although they may be provided with free accommodation by the bar owners. Even if they were paid, earning between 500–1000 Ugandan shillings (US$0.25–0.50) per day, they may have had to supplement their income in order to support themselves. For some women they got this support through tips from customers:

They get money in a way that every man who comes wants to buy them a beer. So they do not drink it all, they just keep money aside and in one day one can make over 20,000 shillings. While others just splash money to get the girl after getting drunk. You cannot imagine that someone can give a waitress 50,000 shillings just for supper.  (female bar owner)

Or from a regular partner or someone who became a regular partner.

I kept on getting customers wanting to fall in love with me but as you know, they were just talking without being serious not until there was some customer who came determined with 10,000 shillings and gave it to me I had to go in for him and he eventually became my current partner.  (47-year-old woman)

We were told a local saying by one of the female local officials: ‘No woman would resist a man's advances at the lake/landing site.’

Having new female workers in a bar was a way of attracting more customers, and some young women, often influenced by others working at the landing sites, arrived from the mainland to work specifically as bar attendants and got drawn into transactional sex with customers. For various reasons, therefore, some women engaged in transactional sex and could be derided as being ‘just sex workers’ by others, but they did not define themselves as such.

#### Commercial sex work

Although none of the women interviewed identified herself as a ‘sex worker’, at some of the landing sites key informants could identify the lodges where women were known to practise commercial sex. Some women were known to come to the landing sites to make money from sex work, and would move between the different landing sites for this work. A male key informant reported how in the past women would engage in sex work to accumulate enough savings and then leave; however, he stated that the young sex workers today are accumulating cash to survive. This may reflect that as with other activities at the landing sites, the nature of sex work changes depending on competition, as well as the fish catch and daily availability of cash. The price for sex could be as little as 3000–5000 Ugandan shillings (US$1.50–2.50) or up to 15,000–20,000 Ugandan shillings (US$7.50–10.00) for a whole night. The price was lower when a condom was used; many men were willing to pay more for what was termed locally as ‘live sex’ (sex without a condom). In addition to adult men who visited the sex workers, young boys aged 14–18 years frequented sex workers with 3000–5000 Ugandan shillings from the cash they earned in that day, which can be in the range of 5000–10,000 Ugandan shillings from carrying out work at the landing sites, such as packing fish and repairing sacks. As one female key informant said: ‘There is a saying among the youth that, when there is good money in a man's wallet that he has earned for a day, he is going to buy a sex worker.’ These young boys could not afford to sustain relationships on their uncertain and irregular incomes given women's expectations of gifts, but they gained sexual experience from commercial sex.

#### Transactional sex and business

While some transactional sex at the sites appears to be based around ‘commercial sex work’, and bars and lodges, sex is sometimes used in transactions at the lakeshore to establish working relationships, with fishermen demanding sex before selling fish. However, the relationship between the role of a sexual partner and economic opportunity is often not as clear cut as this suggests.

The role of gifts and support in relationships is apparent in many narratives with women often citing the need for support or frustrations at the lack of support they were receiving. For some women their partner did not just support with gifts, money or food, but supported the woman in setting up a business especially in fish processing – by going into business together, or giving the woman the initial capital she needed, or supplying fish. For example, the husband of one 36-year-old woman would bring back small silver fish and vegetables that she would sell on. Slowly she started to purchase fish from other fishmongers. In this case she did not continue to rely solely on her partner for access to commodities. It is clear that her relationships played a role in the line of business that she went into; however, she later resolved not to have sexual relationships with men in order to get support but to support herself independently.

There is often friction in the need for support and the desire for independence. One significant reason given for ending a relationship was that the partner did not provide this ‘support’. A 41-year-old woman who had a house reported that:

I would like to remarry so as to gain support, but once you get a sexual partner while you are in your own house, that partners may have the wrong idea about such a woman, assuming that because she owns a house she has money as well. Then you find that he does not give you any support, instead such a partner with such a bad habit, he consumes all your money without gaining anything out of him. You wonder and regret as to why you got such a partner!

Other women were not able to refuse such relationships. A 27-year-old woman started working as a house-girl and then moved into restaurant work. When the restaurant collapsed she was employed to dig someone's land. She needed help from her partner. He gave her support and despite his promiscuous behaviour she feared losing him as a partner.

Despite the struggles against adversity that many women encountered, some women were in fact very successful, both in acquiring the capital to expand businesses, purchase land and build their own house, and in holding positions within the local councils and fisheries management bodies governing the landing sites. Having successfully gained the independence sought, such women work hard to maintain this, and did not want this position threatened through their relationships with partners.

He was too much of a drunkard and he could not give me anything at all! He thought that because I was working and owning a house that he was in need of financial assistance instead! I decided to abstain till date.  (27-year-old female market vendor)

However the majority women were still subordinate to men in their relationships both professionally and personally. This was reflected in their bargaining power in transactions as well as in sexual relationships, and impacted on the decisions that the women made, putting them in perhaps more vulnerable positions in terms of violence and sexually transmitted infections. They may be unable to negotiate safer sexual practices or a test for HIV infection with the man. For example, a 19-year-old woman had decided that she did not want children with her current partner because ‘of his ways’ (promiscuity); however, she did not feel able to discuss contraception, such as condom use, with him and therefore opted for an injection (of hormonal contraception) so she would not become pregnant, but that injection could not protect her from sexual transmitted infections. Another woman, aged 28 years, stated that she could not insist on condoms from her partner as he was giving her support to run her restaurant. We have discussed some of these challenges elsewhere.^32^

## Discussion

The ability of women to create a viable livelihood at the landing sites is influenced by a wide variety of factors. However, the case studies presented here suggest strongly that women who had or were able to access capital when they arrived at the landing site to set up their own enterprise had a significant advantage over those who did not. For some women this capital came from friends or other family members, whilst for others it was from husbands and partners. Similarly women that had particular skills, for example, tailoring or had another source of capital were more likely to be able to engage in profitable enterprises. This allowed them to avoid lower paid and more risky work such as fish processing and selling or working in bars. However, the ability of the women and the resources that they had did not necessarily protect them from occurrences such as theft, ill-health and frequent pregnancy that interrupted the work of many of the women interviewed. However, not surprisingly, women who were well supported or had assets were more able to return to productive enterprise once the interruption had passed.

Although the role of friends and family was significant for some women, the importance of support from partners came out very strongly in the women's narratives. Although the risks of sex for fish and commercial sex are well reported, within established partnerships the transactional element of the relationship often compromises women's ability to avoid the potential risks of unprotected sex resulting in HIV infection, other sexually transmitted infections and pregnancy. The support of partners, who are themselves in a vulnerable economic situation, was also shown to be often unreliable and inconsistent, limited by the changing fish stocks and competition in the fisheries. Moreover, some more successful women indicated that success itself exposes women to another risk which arises from gender inequality – that the transactional component of the relationship is reversed and men expected support from the woman. It can then prove difficult for a woman to leave that relationship.

As men and women continue to be drawn to the landing sites in search of opportunities, fears have been raised that women are increasingly being pushed out of the activities in which they traditionally engaged.^33^ Women in this study who were engaged in the most profitable activities were involved in those not related to fishing, which may be an indication that the more profitable areas of processing and trading were becoming less accessible to them. A growing awareness of the urbanization and growth in leisure industries at landing sites^34^ may mean that women are now drawn to the landing sites primarily to engage bar and restaurant work. Whilst this opens up further opportunities for the women to capture income from the fisheries, it also exposes them to the potential risks inherent in such work.

The findings show the tension between women's desire for economic independence and the important contribution that economic and practical support from family, friends and partners can make to women's livelihood endeavours at the landing site. While reliance on partners for support may be essential, it can expose women to a wide variety of risks. Whilst transactional sex is not unique to fishing communities, the social and economic characteristics, including the unpredictable cash income, of landing sites means that the risks women face may be greater and the incentives to engage in transactional sex, for short-term financial gains and longer-term support, are more pronounced than in some other settings.

## Notes

1. Porter et al., “Globalization and Women in Coastal Communities”; Williams, “Why Look at Fisheries.”

2. Westaway et al., “Feckless and Reckless or Forbearing and Resourceful?”; Kissling et al., “Fisherfolk are among Groups Most at Risk.”

3. Béné and Merten, “Women and Fish-for-Sex”; Luke, “Age and Economic Asymmetries.”

4. Tindall and Holvoet, “From the Lake to the Plate.”

5. Medard and Wilson, “Changing Economic Problems for Women”; Béné and Merten, “Women and Fish-for-Sex”; Bennett, “Gender, Fisheries and Development”; Geheb et al., “Nile Perch and the Hungry”; Lwenya and Yongo, “Fisherman's Wife”; Medard, “Relations between People.”

6. Mojola, “Fishing in Dangerous Waters,” p. 152.

7. Geheb et al., “Nile Perch and the Hungry.”

8. Medard, “Relations between People.”

9. For example, Ogutu-Ohwayo, “Changes in the Prey Ingested.”

10. Madanda, *Commercialisation and Gender Roles*.

11. Madanda, *Commercialisation and Gender Roles*, p. v.

12. Merten and Haller, “Culture, Changing Livelihoods and HIV/AIDS.”

13. Béné and Merten, “Women and Fish-for-Sex,” p. 889.

14. Kwena et al., “Transactional Sex in Fishing Communities.”

15. Mojola, “Fishing in Dangerous Waters,” p. 150.

16. Pickering et al., “Sexual Behaviour in a Fishing Community.”

17. Wamoyi et al., “Women's Bodies Are Shops.”

18. Obbo, “Dominant Male Ideology and Female Options,” p. 385.

19. Davis, “On the Sexuality of ‘Town Women’.”

20. Godfrey, “Household Gender and Resource Relationships,” p. 19.

21. Davis, “On the Sexuality of ‘Town Women’.”

22. Kuhanen, “Sexualised Space, Sexual Networking.”

23. Asiki et al., “HIV and Syphilis Prevalence”; Seeley et al., “High HIV Incidence.”

24. Asiki et al., “HIV and Syphilis Prevalence.”

25. Boyatzis, *Transforming Qualitative Information*.

26. Pringle, “Origins of the Nile Perch”; Seeley et al., “Fishing for a Living.”

27. Beuving, “Playing Pool along the Shores.”

28. Asiki et al., “HIV and Syphilis Prevalence.”

29. Obbo, “Dominant Male Ideology and Female Options,” p. 382.

30. Allison and Seeley, “HIV and AIDS among Fisherfolk”; Tindall and Holvoet, “From the Lake to the Plate”; Béné and Merten, “Women and Fish-for-Sex.”

31. Wolff et al., “Women Who Fall by the Roadside”; Nyanzi et al., “Mobility, Sexual Networks and Exchange”; Allison and Seeley, “HIV and AIDS among Fisherfolk.”

32. McArthur et al., “How HIV Diagnosis and Disclosure.”

33. Medard, “Relations between People.”

34. Beuving, “Playing Pool along the Shores.”
